# Mechanisms of Short-Chain Fatty Acids Derived from Gut Microbiota in Alzheimer's Disease

**DOI:** 10.14336/AD.2021.1215

**Published:** 2022-07-11

**Authors:** Xiao-hang Qian, Ru-yan Xie, Xiao-li Liu, Sheng-di Chen, Hui-dong Tang

**Affiliations:** ^1^Department of Neurology and Institute of Neurology, Rui Jin Hospital, Shanghai Jiao Tong University School of Medicine, Shanghai 200025, China.; ^2^Shanghai Guangci Memorial hospital, Shanghai 200025, China.; ^3^Department of Neurology, Shanghai Fengxian District Central Hospital, Shanghai Jiao Tong University Affiliated Sixth People’s Hospital South Campus, Shanghai 201406, China.

**Keywords:** Alzheimer's disease, gut microbiota, short-chain fatty acids

## Abstract

Short-chain fatty acids (SCFAs) are important metabolites derived from the gut microbiota through fermentation of dietary fiber. SCFAs participate a number of physiological and pathological processes in the human body, such as host metabolism, immune regulation, appetite regulation. Recent studies on gut-brain interaction have shown that SCFAs are important mediators of gut-brain interactions and are involved in the occurrence and development of many neurodegenerative diseases, including Alzheimer's disease. This review summarizes the current research on the potential roles and mechanisms of SCFAs in AD. First, we introduce the metabolic distribution, specific receptors and signaling pathways of SCFAs in human body. The concentration levels of SCFAs in AD patient/animal models are then summarized. In addition, we illustrate the effects and mechanisms of SCFAs on the cognitive level, pathological features (Aβ and tau) and neuroinflammation in AD. Finally, we analyze the translational value of SCFAs as potential therapeutic targets for the treatment of AD.

Alzheimer’s disease (AD) is the most common cause of dementia, and it is the sixth leading cause of death worldwide [1]. According to the World Alzheimer Report, there were more than 50 million dementia patients worldwide in 2018, and the number is expected to rise to 150 million by 2050 [2]. AD is a progressive neurodegenerative disease with clinical symptoms that range from mild spontaneous cognitive impairment at the early stage to severe neurological and psychiatric symptoms at the advanced stage, including executive function, complex attention, and language dysfunction [3,4]. The known neuropathological features of AD include extracellular neuritic plaques of massed β-amyloid (Aβ) proteins, intraneuronal neurofibrillary tangles that aggregate hyperphosphorylated tau proteins, gliosis, and neuronal loss [5-7].

Currently, an early-life low level of education; midlife hypertension, hearing loss, obesity, head trauma, and intemperance; and later-life depression, antisocial behavior, smoking, lack of physical exercise, air pollution, and diabetes, are identified as the risk factors most closely associated with AD [8]. Evidence has shown that an imbalance of gut microbiota caused by genetic and environmental factors greatly contributes to the progression of AD [9]. For example, changes in gut microbiota diversity have been found in patients with AD and mild cognitive impairment, as compared to healthy individuals [10]. In addition, transplantation of gut microbiota from healthy mice into the intestinal tract of AD model mice was shown to ameliorate the cognitive impairment of AD mice and reduce pathological changes such as Aβ plaques, tau hyperphosphorylation, and neuroinflammation [11]. These discoveries have provided new strategies for the early diagnosis and treatment of AD. However, the mechanism by which gut microbiota and the brain communicate is not clear and may involve several systems, including the peripheral nervous system (vagus nerve and enteric nervous system), metabolic system, endocrine system, and immune system [12-14].

A wide variety of microorganisms colonize the human gastrointestinal tract, including bacteria, viruses, archaea, eukaryotic microbes, and bacteriophages [15]. There are more than 1000 microbial species and approximately 10^14^microorganisms, which is more than 100 times the number of human body cells [16]. In addition, the human microbiota has more than 4 × 10^6^genes, while the number of human genes is 26000 [17,18]. Bacteria constitute the greatest proportion of microbes in the human intestinal microbial system, especially *Firmicutes* (about 51%) and *Bacteroidetes* (about 48%) [16,19]. The microbiota secrete a variety of metabolites that participate in human growth, development, and pathological processes, including short-chain fatty acids (SCFAs), bile acids, and neuroactive molecules [15,20]. SCFAs are the most abundant metabolites derived from the metabolism of indigestible dietary fibers by gut microbes. Recently, SCFAs have been associated with a variety of human diseases such as obesity, diabetes, and neurodegenerative diseases [20,21].

This review focuses on the mechanism of action of SCFAs in AD. The review begins with the metabolism, distribution, and mechanism of SCFAs. The evidence of a correlation between SCFAs and AD is then summarized. Subsequently, the effects and mechanisms of SCFAs on cognitive impairment, pathological changes, and neuroinflammation in AD are analyzed. Finally, the application of SCFAs as targets in AD treatment is discussed to provide a theoretical basis for the further study of SCFAs.

## 1. Biochemical and functional features of SCFAs

### 1.1 The metabolism and distribution of SCFAs

SCFAs are saturated fatty acids with less than six carbon atoms, including formic acid, acetic acid, propionic acid, butyric acid, and valeric acid. In humans, SCFAs are produced mainly through anaerobic digestion of dietary fiber or indigestible carbohydrates by microorganisms in the colon [22]; small amounts of SCFAs are formed from peptide, protein, and glycoprotein precursors [23,24]. Although most types of SCFAs can be generated in the colon, the main SCFAs are acetic acid, propionic acid, and butyric acid, accounting for approximately 60%, 20%, and 20%, respectively [25]. The production of SCFAs is followed by absorption into the mucous epithelium of the cecum and colon, which is a very effective process that absorbs approximately 90-95% of the total yield [26,27]. Once it is absorbed, colonic epithelial cells use butyrate as a metabolic substrate to supply energy for themselves, accounting for 60-70% of the energy requirement of isolated colonic epithelial cells [28]. The remaining SCFA anions are transported through volume-regulated anion channels, driven by Na^+^ efflux [29], to the portal system and then to the liver. Hepatocytes undergo gluconeogenesis using the remaining propionic acid and butyric acid, and 50-70% of acetate is taken up for cholesterol and fatty acid synthesis [30,31]; this leads to circulating concentrations of propionate and butyrate 1-15 µM and acetate concentrations of 100-200 µM [28,32,33]. The other major metabolic site for SCFAs is muscle cells, which generate energy using acetate [30]. Only small amounts of colon-derived SCFAs (approximately 36% acetic acid, 9% propionic acid, and 2% butyric acid) are present in the bloodstream and transported to other tissues throughout the body; these mediate a wide range of biological functions, including host metabolism, immunity regulation, and appetite regulation [34,35].

There is some question as to whether all SCFAs can cross the blood-brain barrier (BBB). In 1973, Oldendorf et al. demonstrated in rats that SCFAs other than formic acid can cross the BBB, as measured by the brain concentration of^14^C-labeled SCFAs injected into the carotid artery [36]. The BBB penetration efficiency was the highest for butyrate, followed by propionate; the lowest for acetate. All of these showed feedback inhibition on brain uptake [36]. In 1979, Bachmann found that the human brain concentrations of propionate and butyrate are 9.4-13.5 nmol/500 mg and 6.7-8.5 nmol/500 mg, respectively, using gas chromatography [37]. In addition, studies have shown that mice treated with live *Clostridium butyricum* have increased levels of butyric acid in the brain [38,39]. Although SCFAs can pass through the BBB, the concentrations of SCFAs are very limited. Recently, SCFAs in the central nervous system (CNS) were documented as regulating the formation of the BBB, microglial maturation, and synaptic plasticity [40-42].

### 1.2 Mechanism of SCFA signal transduction

#### 1.2.1 Ligands for G protein-coupled receptors (GPRs)

To date, six SCFA receptors have been identified: GPR41 (free fatty acid receptor 3, FFAR3), GPR42 (G protein-coupled receptor 42), GPR43 (FFAR2), GPR109A (hydroxycarboxylic acid receptor 2, HCAR2), GPR81, GPR164 (olfactory receptor family 51 subfamily E member 1, OR51E1), and OR51E2 (Olfr78) [25]. Among them, GPR41, GPR43, and GPR109A were discovered earlier and have been studied more comprehensively; the GPR41, GPR43, and GPR109A receptors are widely expressed in humans [39,43]. High expression levels of GPR43 have been detected in immune cells, especially neutrophils and monocytes [44,45], indicating an important role in the regulation of immunity. The others are expressed in the distal colon, skeletal muscle, heart, and liver [46-48]; GPR43 is not found in the CNS or peripheral nervous system. GPR41 expression has been detected in adipose tissue, immune tissue, and the liver [44-46]; it is also located in autonomic and somatic sensory nerve cell bodies [49], the sympathetic nervous system [50], and nerve fibers of the portal vein [51]. Moreover, the expression of GPR41 and GPR43 changes in different physiological or disease states; for example, the concentration of GPR43 in the rat proximal colon is upregulated by increases in the uptake of indigestible carbohydrates [52]. In diet-induced obesity models, the transcription of GPR43 is upregulated in adipose tissue, the liver, and some of the skeletal muscles [53-55]. GPR109A, a nicotinate receptor, was shown to be activated by butyrate and h-D-hydroxybutyrate with an 50% effective concentration (EC50) of 1.6 mmol/L [56,57], and can be expressed in white or brown adipose tissue, immune cells, and epithelial cells in the small and large intestines [58]. Notably, the expression of GPR 109A has been detected in the brains of mammals, including the hypothalamic neurons in rodents [59] and the rostral ventrolateral medulla [60].

**Table 1:** The expression sites, ligands, signaling pathways, and functions of SCFA receptors.

Receptor	Localization in the body (nervous system)	SCFAs substrate	Physiological function	References
**GPR41 (FFAR3)**	adipose tissue, lymph nodes, pancreas, spleen, bone marrow, peripheral blood mononuclear cells, colon, small intestine (peripheral nervous system, nerve fibers of the portal vein, vagal, dorsal root, and trigeminal ganglia)	propionate, butyrate, valerate > acetate > caproate	• regulation of intestinal gluconeogenesis • suppressor of appetite • regulation immunity and inflammation • gastrointestinal functionality • regulation sympathetic nervous system • protecting the blood-brain barrier	[25,45,46,50,51,72-74]
**GPR43 (FFAR2)**	neutrophils, monocytes, peripheral blood mononuclear cells, B-lymphocytes, polymorphonuclear cells, eosinophils, skeletal muscle, heart, adipose tissue, distal ileum and colon, small intestine	acetate, propionate > butyrate > valerate > formate	• cholesterol/lipid metabolism • the immune response • suppressor of appetite • gastrointestinal functionality • carcinogenesis; • regulation the metabolism	[25,45,46,51,73,74]
**GPR109A (HCAR2)**	adipocytes, monocytes, macrophages, neutrophils, dendritic cells and epidermal Langerhans cells, retinal pigment epithelium, the intestinal epithelium, (rostral ventrolateral medulla, PC12 cells, hypothalamic neuron)	butyrate, 3-hydroxybutyrate	• regulation of lipid and immunity; • as a tumor suppressor; • cellular effects in the epidermis; • bone remodeling	[56-58,75,76]
**GPR164 (OR51E1)**	heart, epicardial adipose tissue, prostate tissue, tubule system of kidney tissue, gastrointestinal mucosae	butyric acid	• regulation of cardiac function; • modulators of the renal physiology; • gastrointestinal enteroendocrine activity	[77-81]
**OR51E2 (Olfr78)**	kidney blood vessel, prostate cancer, epidermal melanocytes, (autonomic nervous system cells)	acetate, propionate	• modulation blood pressure; • involvement in tumor process	[82-84]

All GPRs transduce signals by activating downstream G proteins, including the α, β, and γ subunits [46]. The Gα subunits can be grouped into four subclasses (Gαi, Gαq, Gαs, and Gα12) [46] and are linked to various mitogen-activated protein kinases (MAPKs), such as p38MAPK, JNK, and ERK1/2, which transduce the signal [61]. Due to the complexity of the downstream pathway of GPRs, SCFA-receptor binding produces complex signal transduction mechanisms and biological effects. Gαi/o can be coupled with GPR41 and GPR43, whereas Gαq can be coupled only with GPR43 [62]. Coupling of the two receptors induces inositol 1, 4, 5-trisphosphate formation, an intracellular calcium increase, ERK1/2 activation, and a decrease in cyclic adenosine monophosphate (cAMP) [44,63,64]. The coupling of acetate-activated GPR43 to ERK1/2 is weaker than that of propionate-stimulated GPR41 [65]. In addition, the activation of ERK1/2 by GPR41 and GPR43 is different: GPR41 acts through PI3K, and GPR43 activates ERK1/2 through Src without the activation of Raf-1 [65]. Transfection of either GPR41 or GPR43 into HEK293 or CHO-K1 cells can result in weak activation of the JNK and p38MAPK pathways [65]. Increased phosphorylation of p38 was also observed in MCF-7 cells treated with a GPR43 agonist [66]. Furthermore, recent studies have reported that the G (i/o) βγ pathway and β arrestin2 can be coupled by GPR43 [67-69]. GPR109A is sensitive to pertussis toxin, indicating that the receptor couples to the Gi protein [70,71]; activation of the Gi protein leads to the inhibition of adenylyl cyclase in most cell types. It can also activate the β-isoforms of phospholipase C through Gβγ subunits in some cells, especially in the immune system [57]. The expression distribution, ligands, signaling pathways, and functions of the other receptors are shown inTable 1.

#### 1.2.2 Inhibition of histone deacetylation

Epigenetic regulation, including DNA methylation, chromatin remodeling, non-coding RNA regulation, and histone modifications, has been shown to play a crucial role in the growth and development of the nervous system and neurodegenerative diseases such as Huntington’s disease, Parkinson’s disease, amyotrophic lateral sclerosis, and AD [85-87]. Nucleosomes, made up of 147 base pairs of DNA surrounded by double-copy histones (H2A, H2B, H3, and H4), are the basic building blocks of chromosomes [86,88]. Nucleosome regulation of gene expression involves two main mechanisms: ATP-dependent remodeling of chromatin complexes, which results in rapid chromosomal rearrangements [89], and post-translational modification of histones at more than 20 possible sites [90], including acetylation, SUMOylation, methylation, ubiquitylation, and phosphorylation [88,91]. Among these, histone acetylation is one of the most important epigenetic mechanisms in the development of AD and is a bridge between environmental and genetic factors [88,91,92]. The status of histone acetylation is the result of the dynamic activities of two kinds of enzymes with opposing functions: histone acetyltransferases (HATs) and histone deacetylases (HDACs) [93]. Histones are acetylated by HATs; this loosens the chromatin structure and makes it easier for transcription factors to bind to gene promoters and affect transcription. HDACs act in the opposite direction; they remove acetyl groups from histones [94]. In mammals, HDACs are divided into four classes based on their structure, function, and subcellular localization [91].

HDAC inhibitors are divided into four classes based on their molecular structures: aliphatic acids, hydroxamates, benzamides, and cyclic peptides [95]. SCFAs belong to the aliphatic acid class and act as broad-spectrum inhibitors of HDAC enzymes in the millimolar range [96]. Class I HDACs, class II HDACs, and some class III HDACs can be non-competitively inhibited by butyrate and propionate [97-99]. Valeric acid may be another inhibitor of class I HDACs [100]. The inhibitory efficiency of butyrate is about 80%, which is the highest among SCFAs; propionate has approximately 60% inhibitory efficiency [101]. SCFAs play a wide range of roles in different cells and tissues by inhibiting the action of HDACs; for example, the SCFAs methoxyacetic acid and valproic acid can improve the transcriptional efficacy of nuclear hormone receptors, such as estrogen and progestin nuclear hormone receptors, to increase cellular sensitivity [102]. In the immune system, propionate and butyrate promote the apoptosis of neutrophils through HDAC inhibition by a mechanism not involving GPRs and MAPKs [103]. In addition, n-butyrate can regulate the function of macrophages in the intestines by inhibiting HDACs instead of through toll-like receptor signaling and activation of GPRs [104]. The signal transduction mechanisms of SCFAs are summarized inFigure 1.

## 2. SCFAs and AD

Studies have detected variations in gut microbiota diversity in AD patients and mouse models [10,105]; these caused changes in the concentrations of SCFAs. In 2017, Yilmaz et al. compared metabolite concentrations in saliva samples between AD patients and healthy controls using^1^H-NMR metabolomics, and they found an increased level of propionate in the AD patients [106]. Similarly, another study reported that propionate and acetic acid concentrations in saliva samples from AD patients were 1.35 and 1.25 times higher, respectively, than those in the control group [107]. However, a recent study found that the serum of AD patients had lower concentrations of acetate, which was associated with a higher risk of AD [108]. In fecal samples, seven SCFAs (formic acid, acetic acid, propionic acid, butyric acid, 2-methylbutyric acid, isovaleric acid, and valeric acid) showed progressively decreased levels among healthy controls, patients with amnestic mild cognitive impairment, and AD patients [109].

In APP/PS1 transgenic mice, which are a model of AD, the concentrations of butyric acid and isobutyric acid were both decreased in the feces and brain [110]; butyric acid concentrations in the brain tissue were positively correlated with those in the feces [110]. Another study found lower levels of propionic acid and higher levels of lactic acid in the APP/PS1 mice compared to wild-type mice, as measured in the feces through stable isotope labeling and liquid chromatography-tandem mass spectrometry joint analysis [111]. The differences in the SCFA concentrations in the AD and wild-type mice existed whether they were the same age or at different life stages. For example, the SCFAs were significantly decreased in 11-month-old 3xTg-AD mice compared with both 3- and 6-month-old 3xTg-AD mice; this trend occurred much later than observed in wild-type mice (6 months) [112]. In addition, the concentrations of acetate were dramatically decreased in AD-model *Drosophila*, accompanied by a reduced abundance of *Acetobacter* and *Lactobacillus* [113]. Among the published reports, the concentration of SCFAs varies significantly in patients with AD, but the differences in test samples lead to inconsistent results from one study to another [10,114,115].

**Figure 1.**
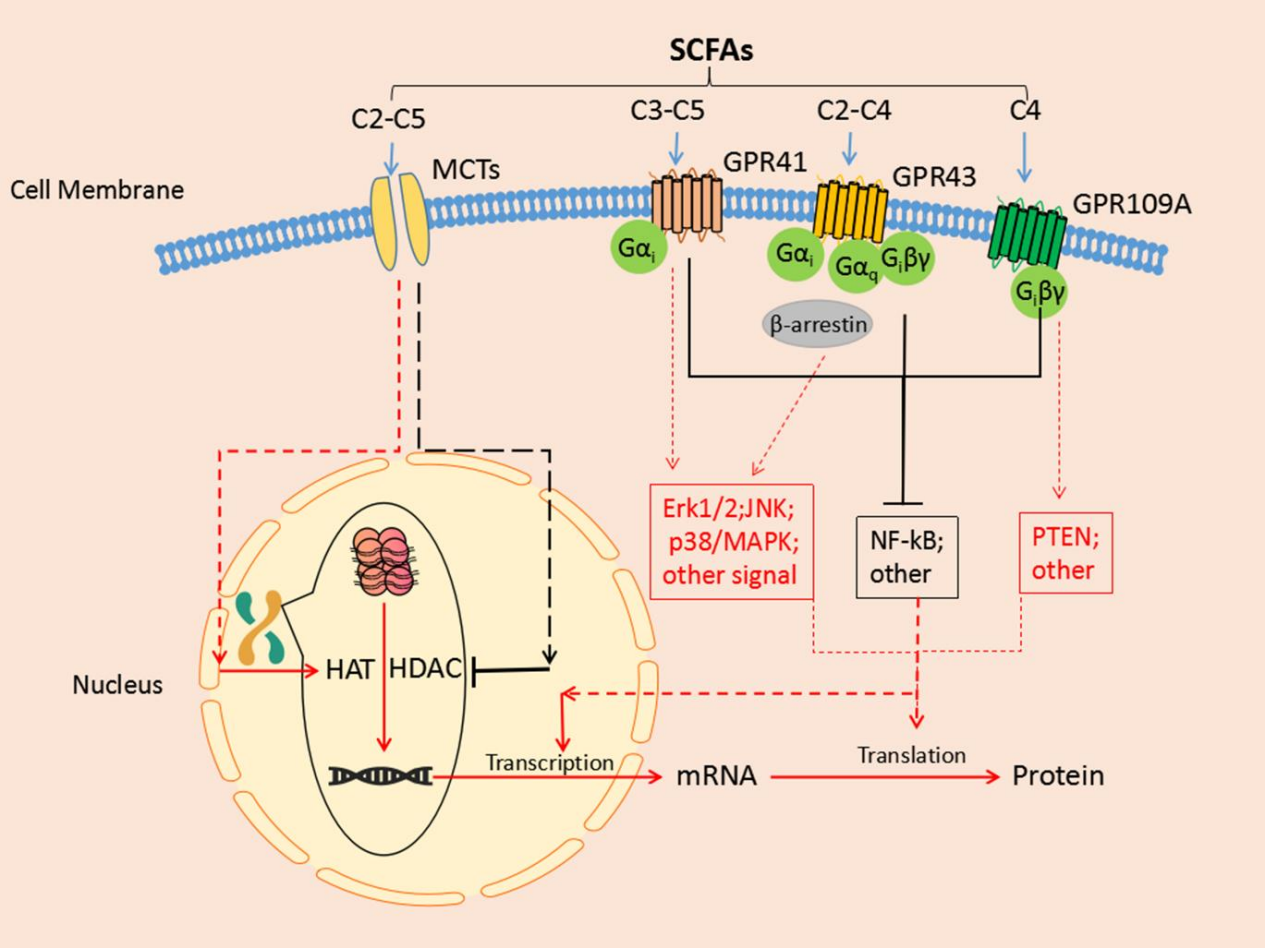
**An integral view of the cellular signal transduction pathway of SCFAs**. Short-chain fatty acids (SCFAs) affect biological functions through two main pathways. They can bind G protein-coupled receptors on the surface of cell membranes (including GPR41, GPR43, and GPR109) to activate downstream NF-κB, MAPKs, and other signaling pathways. They can also enter cells through MCTs on the cell surface and participate in inhibiting HDACs or promoting HATs to regulate gene transcription. GPR, G protein-coupled receptor; NF-κB, nuclear factor-κB; MAPKs, mitogen-activated protein kinases; MCTs, monocarboxylate transporters; HDACs, histone deacetylases; HATs, histone acetyltransferases

### 2.1 SCFAs and cognitive impairment

As the most representative clinical symptom of the early stage of AD, cognitive impairment has always been a focus of clinical and basic research on AD. SCFAs have been demonstrated to ameliorate cognitive impairment caused by AD or a variety of other factors, such as isoflurane exposure, scopolamine, and radiation [116-119]. While sodium butyrate has no effect on learning and memory in normal wide-type rats [117], it can ameliorate the cognitive impairment of the early and advanced stages of AD in an AD mouse model [119-121]. Regarding the potential mechanism by which SCFAs improve cognition, Lee et al. discovered that sodium butyrate reversed radiation-induced downregulation of phosphorylated cAMP response element binding protein (p-CREB)/brain-derived neurotrophic factor (BDNF) expression [116]. Analogously, experimental pneumococcal meningitis-associated memory impairment can be improved by sodium butyrate through increasing the expression of BDNF and glial cell line-derived neurotrophic factor [122]. In an AD mouse model, Govindarajan et al. found that sodium butyrate amelioration of cognitive impairment was associated with an increase in the expression of memory-consolidation genes, such as MYST4, Marcksl1, GluR1, SNAP25, and SHANK3 [120]. In addition, butyrate was shown to improve synaptic plasticity in 8-week-old 5xFAD mice by increasing synapse-associated proteins and promoting long-term potentiation and depotentiation [123]. In microglia, sodium butyrate can upregulate the PI3K/AKT/CREB/BDNF signaling pathway, which contributes to long-term potentiation and synaptic plasticity [124]. In neural regeneration, physiological levels (µM) of SCFAs can promote the mitosis of human neural progenitor cells by regulating the expression of genes related to proliferation, apoptosis, and neurogenesis [125]. However, SCFAs at millimolar (mM) concentrations are toxic to neural stem cells [125]. The changes in the signaling pathways mentioned above are ultimately related to the activities of SCFAs as HDAC inhibitors and to chromosome remodeling, which ameliorates impairments in cognition and memory [126].

### 2.2 SCFAs and Aβ-tau pathology

The decades-old amyloid hypothesis, currently the most popular hypothesis for AD, is a major focus of current research [127]. In this hypothesis, the abnormal accumulation of extracellular Aβ peptides is considered the core pathological feature of AD. Aβ peptides are derived from the sequential proteolytic cleavage of amyloid precursor protein (APP) by β- and γ-secretase [7]. The tau hypothesis, another hypothesis for AD etiology, is not as specific to AD as Aβ and can be observed in frontotemporal dementia, progressive supranuclear palsy, corticobasal degeneration, and Pick’s disease. [6]. The tau hypothesis postulates that tau pathology arises first in a specific brain region (the entorhinal cortex or locus coeruleus) and then travels along the nerve loop to the entire cerebral cortex, leading to neurodegeneration [128].

Studies have shown that SCFAs have a regulatory effect on both Aβ and tau pathologies. Clinical studies have shown that Aβ levels in patients with AD were positively correlated with the serum concentrations of acetate and valerate and negatively correlated with the level of butyrate [129]. SCFAs can alter the pathological effects of Aβ in several ways. Treatment with oral sodium butyrate shows a dose-dependent reduction in Aβ levels in the brains of 5xFAD mice at the early stage of disease progression [119]. However, injection of sodium butyrate into the lateral brain ventricles of APP/PS1-21 mice at an advanced disease stage does not reduce Aβ levels [120]; this may be related to the administration, dosage, and duration of sodium butyrate treatment. The spontaneous aggregation of monomeric Aβ into more neurotoxic Aβ oligomers or Aβ fibrils is an important pathological process in the brains of patients with AD. The SCFAs propionic acid, butyric acid, and valeric acid were observed to inhibit the aggregation of both monomer Aβ_1-40_ and Aβ_1-42_ into Aβ oligomers and Aβ fibrils [130]. Moreover, sodium butyrate was shown to promote mitochondrial function and cell proliferation, which ameliorates Aβ-induced N2a cell damage [131]. In addition, sodium propionate showed a protective effect against Aβ-induced neurotoxicity by inhibiting the production of inducible nitric oxide synthase and cyclooxygenase-2 (COX-2) [132]. In amyloidogenic APP processing, sodium butyrate can decrease the expression of APP and increase NEP expression levels [131]. However, sodium butyrate has also been shown to enhance neuronal apoptosis induced by the APP C-terminal fragment (C31, AICD, and C99) by lowering histone deacetylation to regulate gene transcription [133]. Recent preclinical studies have demonstrated that SCFA supplementation (25.9 mM sodium propionate, 40 mM sodium butyrate, and 67.5 mM sodium acetate) increased Aβ plaque deposition in germ-free APP/PS1 mice [134]; there were no significant differences in the expression levels of APP, PS1, BACE1, and ADAM10, or the aggregation kinetics of Aβ, and only a slightly reduced ratio of C83 and C99 [134]. Sodium butyrate can also modulate reactive oxygen species levels mediated by nuclear factor erythroid 2-related factor 2 stabilization to reduce BACE1 expression and Aβ accumulation caused by high cholesterol in SK-N-MC cells [135].

SCFAs have also been associated with tau hyperphosphorylation, another pathological feature of AD. In the forebrain of PS-1/PS-2 conditional double-knockout mice, the level of tau hyperphosphorylation (Ser-199 and Ser-202) was significantly decreased after treatment with sodium butyrate [136]. However, Nuydens et al. reported that treatment of the human neuroblastoma TR14 cell line with sodium butyrate can induce aberrant tau phosphorylation, which may be related to the regulation of cytoskeletal proteins [137].

### 2.3 SCFAs and neuroinflammation

In the last decade, extensive research has shown that neuroinflammation plays an important role in the occurrence and development of neurodegenerative diseases. In AD, neuroinflammation is induced partially by the pathology of Aβ and tau proteins, and there is a close interaction between them [138]. The main sites of neuroinflammation are microglia and astrocytes in the CNS [139]. Microglia are cells of the CNS innate immune system and are widely distributed throughout the brain. In the physiological state, microglia are the main cells that maintain immune homeostasis in the CNS and promote synaptic plasticity [139]. At different stages of AD, resting M0 microglia are activated by Aβ and dead neurons to transform into the M2 subtype of microglia, which have anti-inflammatory and phagocytic functions, or the M1 subtype of microglia that have proinflammatory effects [140]. SCFAs are indispensable for the maturation of microglia and have important regulatory effects on pathological conditions. Erny et al. reported that SCFAs can reverse the damage to microglia caused by having a germ-free gut in mice; this elucidated the role of SCFAs in promoting the maturation and function of microglia [141]. Moreover, in a lipopolysaccharide (LPS)-induced neuroinflammation model, supplying glyceryl triacetate significantly decreases microglial activation and reduces proinflammatory cytokine interleukin-1β (IL-1β) expression at the transcriptional and translational levels by upregulating histone acetylation [142,143]. Butyrate also lowered the secretion of proinflammatory cytokines by microglia and ameliorated the associated neuroinflammation [144]. In an AD model, acetate supplementation can inhibit the ERK/JNK/NF-κB (nuclear factor-κB) pathway to reduce the levels of COX-2 and IL-1β through GPR41 [145]. Moreover, SCFA treatment can promote microglial recruitment to Aβ plaques without influencing the phagocytic capacity of microglia [134].

Recently, Wenzel et al. adopted human THP-1 monocytic cells and differentiated HL-60 myelomonocytic cells to mimic the function of human microglia to explore the effect of SCFAs on the function of microglia. The results showed that SCFAs reduced the secretion of proinflammatory cytokines (IL-1β, MCP-1, and TNF-α) and the phagocytic ability of THP-1 cells; they also suppressed the respiratory burst of HL-60 cells induced by N-formylmethionine-leucyl-phenylalanine [146]. The anti-inflammatory mechanisms of SCFAs are related to the following aspects: 1) SCFA (acetate) supplementation rescues LPS-induced upregulation of phospholipase C β1, COX-1 and COX-2 [147], 2) the expression of COX-2 is inhibited by butyrate in Aβ-induced BV2 cells, accompanied by a lower level of NF-κB-p65 phosphorylation [148]. Moreover, protein kinase B (AKT)-Rho GTPase signaling has been shown to mediate sodium butyrate effects on microglial process elongation [149]. The regulation of SCFAs on the expression level of these pathways ultimately depends on the inhibition of HDACs, which facilitates combination of acetylated H3K9 with the promoter regions of target genes [111,149].

**Figure 2.**
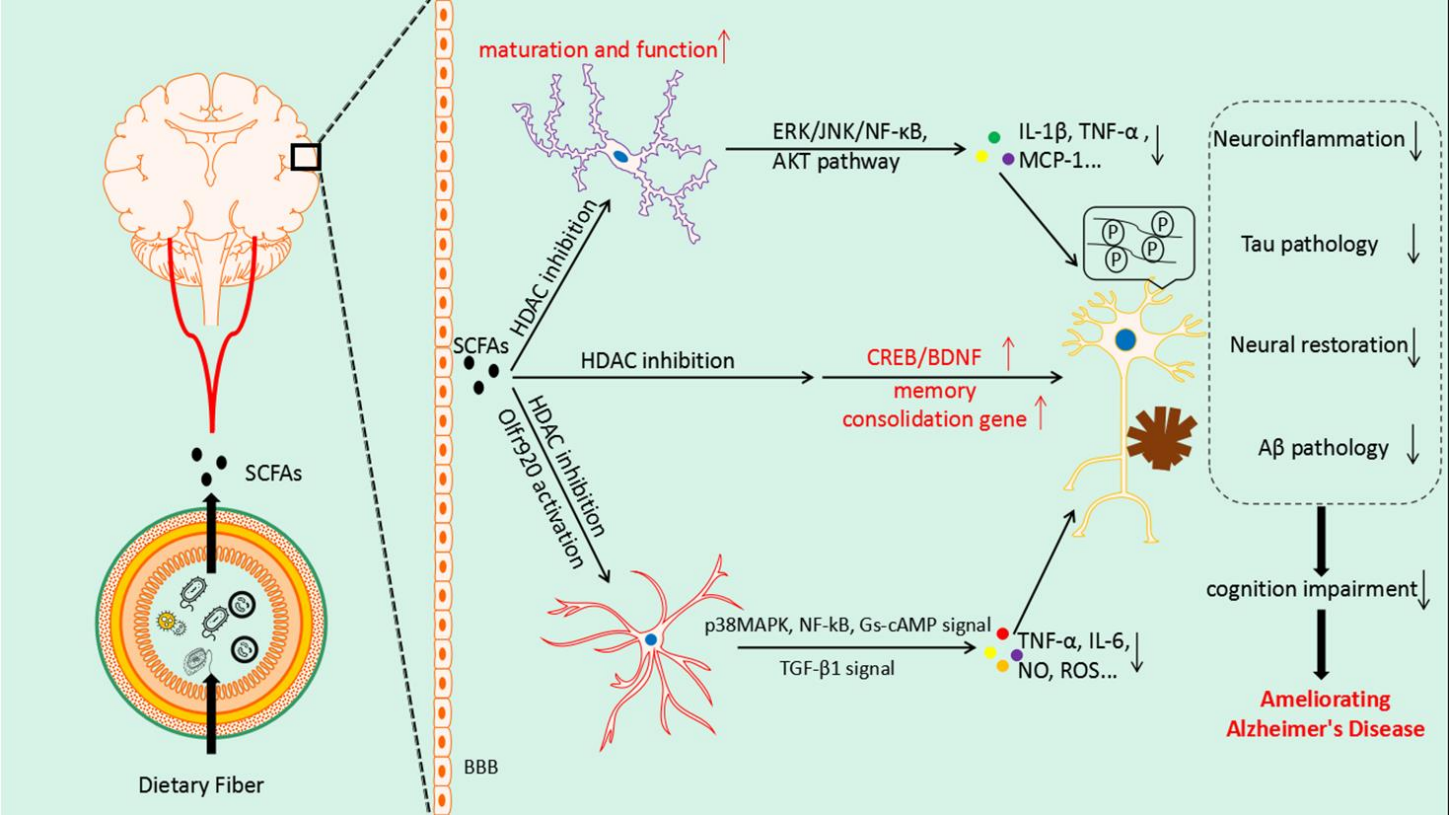
**Overview of the effects of SCFAs in Alzheimer's disease**. SCFAs from the gut microbiota enter the CNS by crossing the blood-brain barrier. They act on neurons to promote neuronal repair and regeneration through upregulation of the CREB/BDNF signaling pathway and expression of memory-consolidation genes. In addition, the secretion of inflammatory factors is reduced by inhibiting the MAPK, NF-κB, and other pathways in disease-related microglia and astrocytes with proinflammatory effects. SCFAs also participate in the pathological regulation of Aβ and tau proteins, ultimately ameliorating cognitive impairment in AD. CREB, cyclic-AMP response element binding protein; BDNF, brain-derived neurotrophic factor; NF-κB, nuclear factor-κB; MAPK, mitogen-activated protein kinase

Astrocytes, a type of CNS glial cell, are important in maintaining CNS homeostasis by regulating neurotransmitter secretion, modulating synapse and BBB function, and providing trophic support for neurons [150,151]. Being similar to microglia, the astrocytes transit from a resting state to the A1 reactive state to promote the pathological process of AD in response to stimulation by inflammatory cytokines from microglia and by Aβ [151]. Several independent studies have demonstrated that SCFAs inhibit the proinflammatory action of astrocytes in multiple ways. First, acetate supplementation can decrease levels of proinflammatory cytokines (TNF and IL-6) by downregulating p38MAPK and NF-κB signaling and increasing anti-inflammatory cytokine concentrations (IL-4) via upregulating TGF-β1 signaling; these may be associated with enhanced H3K9 acetylation [152]. In addition, the LPS-induced secretion of phospholipase A2 (cPLA2), cPLA2 IIA, and phospholipase C β1 was reduced after treatment with acetate [147]. In 2016, Moriyama et al. found that acetate can rescue LPS-induced effects on nitric oxide and reactive oxygen species production, and on the p38MAPK pathway, in cultured primary rat astrocytes [153]. Recent studies have observed that the expression of the odorant receptor Olfr920 in astrocytes can be activated by SCFAs following the activation of the Gs-cAMP pathway; this decreases the activity of LPS-induced reactive astrocytes [154] (Fig. 2).

### 2.4 SCFAs and the BBB

The BBB is a highly selective semipermeable membrane that plays an important role in maintaining CNS homeostasis [155]. The BBB is composed of cerebral endothelial cells, pericytes, the basement membrane, glial cells (astrocytes, microglia, and oligodendrocytes), and smooth muscle cells [156]. These cells are linked functionally with neurons to form the neurovascular unit [156]. A number of studies have shown that BBB dysfunction is crucial to the onset and development of AD, promoting the production of Aβ and reducing its clearance, activating microglia to accelerate neuroinflammation, and driving oxidative stress and neuronal damage [157]. Studies have shown that GPR41 receptors for SCFAs are expressed in endothelial cells [158], including cerebrovascular endothelial cells [72]. SCFAs are indispensable for the establishment of a normal BBB and in the protection and repair of the BBB during disease progression. For example, the BBB permeability in germ-free mice can be reduced by treatment with sodium butyrate [159]. In addition, BBB disruption and brain edema induced by middle cerebral artery occlusion can be attenuated significantly by valproic acid through suppression of the nuclear translocation of NF-κB, degradation of tight-junction proteins, and induction of matrix metalloproteinase-9 [160]. Therefore, the effect of SCFAs on the BBB in AD warrants further study. The mechanism of SCFAs in AD was summarized inFigure 2

## 3. Potential SCFA-targeting AD treatment strategies

SCFAs significantly improve cognition in AD, the pathologies of Aβ and tau, and neuroinflammation, suggesting that they have potential value in the treatment of AD. At present, the regulation of SCFA concentrations in the body is divided mainly into three types. First, the in vivo concentration of SCFAs can be regulated by oral or intravenous supplementation of SCFAs, as summarized above. The second approach is to rebuild a healthy homeostatic system of gut microbes through fecal transplants or probiotics [161]. For example, oral administration of *Bifidobacterium breve strain A1*, which can produce acetate, significantly ameliorated Aβ-induced cognitive impairment [162]. *Clostridium butyricum*, which produces butyrate, can ameliorate cognitive impairment, reduce Aβ deposition, and inhibit neuroinflammation by reducing both microglial activation and secretion of proinflammatory cytokines [148]. In addition, transplantation of wild-type mouse feces into APP/PS1 mice can improve pathological indicators by increasing the production of SCFAs [163]. The proportions of metabolic substrates that are converted into the corresponding SCFAs can be increased by adjusting the diet, including consuming prebiotics or a healthy diet [164]. It was recently reported in patients with mild cognitive impairment that a modified Mediterranean-ketogenic diet increases fecal propionate and butyrate levels, which are negatively correlated with Aβ-42 [165].

## 4. Conclusion

SCFAs, one of the main metabolites of the gut microbiota, play a vital role in pathophysiological processes. Their biological functions are realized mainly by binding to cell membrane receptors that activate downstream signaling pathways or directly enter the cell to regulate histone deacetylation. Abnormal changes are observed in the concentrations of SCFAs in AD. They are involved in AD processes by regulating synaptic plasticity, Aβ and tau pathologies, and neuroinflammation. SCFAs derived from gut microbiota are potential targets for the treatment of AD, but their clinical applications require further research.
